# The Synergistic Effects of Celastrol in combination with Tamoxifen on Apoptosis and Autophagy in MCF-7 Cells

**DOI:** 10.1155/2021/5532269

**Published:** 2021-07-22

**Authors:** Lijun Wang, Luqun Tang, Chengyun Yao, Chunyan Liu, Yongqian Shu

**Affiliations:** ^1^Department of Oncology, The First Affiliated Hospital of Nanjing Medical University, China; ^2^Department of Radiation Oncology, The Affiliated Cancer Hospital of Nanjing Medical University, Jiangsu Cancer Hospital, Jiangsu Institute of Cancer Research, China

## Abstract

Breast cancer is one of the most common cancers among females and is associated with high mortality and morbidity rates. Several studies have demonstrated that combination treatments with natural products and tamoxifen can improve the sensitivity and cytotoxicity of oestrogen-positive breast cancer cells in response to tamoxifen. Celastrol, a triterpene from traditional Chinese medicine, has been proven to exert significant anticancer effects on various cancers. Our study is aimed at exploring the interactive antitumour effects of celastrol combined with tamoxifen and the potential underlying anticancer mechanisms in MCF-7 cells. The results from MTT assays, isobolographic analyses, and clonogenic cell survival assays revealed that a combination of celastrol and tamoxifen exerted synergistic cytotoxic effects in MCF-7 cells. The results from Annexin V/PI staining and flow cytometry analysis suggested that celastrol enhanced tamoxifen-mediated apoptosis. In addition, exposure to a combination of celastrol and tamoxifen inhibited cell proliferation by causing G1 phase cell cycle arrest. Moreover, the distribution of LC3 was monitored by immunofluorescence, and the changes in the LC3II and P62 levels detected by western blot analysis suggested that celastrol in combination with tamoxifen triggered autophagy. Furthermore, the decrease in p-Akt and p-mTOR in MCF-7 cells, along with the increase in the autophagy marker proteins LC3II and P62, suggested that the Akt/mTOR pathway might be involved in the triggering of cell autophagy by the combination treatment. However, in an MCF-7-implanted nude mouse model, it was possible to detect significantly decreased tumour volumes and tumour weights and decreased p-Akt and p-mTOR protein expression in the celastrol+tamoxifen group. Therefore, our study provides the first evidence that celastrol combined with tamoxifen exerts synergistic anticancer effects by inducing apoptosis and autophagy in MCF-7 cells. Considering the urgent need for novel therapeutic strategies in anticancer therapy, this combinatorial approach is worthy of further investigation.

## 1. Introduction

Breast cancer is one of the most common malignancies and a main cause of cancer-related death among females worldwide [1–3]. According to reports, with changes in lifestyles, the incidence of breast cancer among Chinese women is currently increasing [2]. Although current multidisciplinary therapies, including surgery, chemotherapy, radiotherapy, and molecular-targeted therapy, have shown effects in some settings, hormonal therapy with tamoxifen is now considered the gold standard for preventing tumour recurrence in women with hormone-responsive breast cancer [4, 5].

Tamoxifen is classified as a selective oestrogen receptor modulator (SERM) and is commonly used for endocrine therapy in premenopausal and postmenopausal patients with receptor-positive breast cancer. In breast tissue, tamoxifen, an antagonist of oestrogen receptor *α* (ER*α*), competitively inhibits the binding of oestrogen to ER*α*, which blocks the actions of oestrogen on breast cancer cells [5, 6]. However, 20-30% of tumours are resistant to tamoxifen, leading to endocrine therapy failure [7, 8]. Various studies have revealed that alteration of ER signaling, crosstalk between the ER and GFR (growth factor receptor) network, downregulation of ER, and activation of the PI3K/Akt/mTOR pathway contribute to resistance to tamoxifen [9–11]. Therefore, new strategies, such as combination treatments with natural products or treatments targeting the PI3K/Akt/mTOR pathway, are needed to overcome resistance to tamoxifen or enhance the antitumour effect of tamoxifen.

One of the ultimate goals of translational medicine is to develop new treatment strategies for improving the health of the entire population, and more researchers are focusing on treatment with natural products. Celastrol, a quinone methide triterpenoid isolated from the traditional Chinese medicine “Thunder of God Vine” (*Tripterygiumwilfordii hook* F.), has been used in the treatment of autoimmune diseases, chronic inflammation, and neurodegenerative diseases. Recent studies have shown that celastrol can induce apoptosis in various cancer cells, including lung cancer, colon cancer, and breast cancer, by inhibiting proliferation. It was also reported that the antitumour effect of celastrol results from the modulation of the PI3K/Akt/mTOR and MAPK pathways [12, 13]. Recently, much attention has been drawn to the induction of autophagy induced by celastrol, which may play a vital role in its anticancer effect [14, 15].

Although the effects of celastrol on many types of tumour cells and the effects of tamoxifen in ER-positive breast cancer cells have been studied, the relationship between apoptosis and autophagy in MCF-7 cells treated with a combination of celastrol and tamoxifen has not been addressed. Herein, synergistic effects of a combination treatment on apoptosis and autophagy in MCF-7 cells were proposed.

## 2. Materials and Methods

### 2.1. Cell Culture

Human breast cancer cell line MCF-7 (oestrogen receptor positive) was purchased from the Institute of Biochemistry and Cell Biology, Chinese Academy of Sciences (Shanghai, China). Cells were cultured in DMEM medium (Gibco-BRL, Grand Island, USA) supplemented with 10% fetal bovine serum (FBS), 100 U/ml penicillin, and 100 *μ*g/ml streptomycin at 37°C in a humidified atmosphere containing 5% CO_2_.

### 2.2. Reagents and Antibodies

Tamoxifen was purchased from Tocris (Minneapolis, MN). Celastrol was purchased from Shanghai Hotmed Sciences Co. Ltd. (Shanghai, China), with 98% purity or higher. Dimethyl sulfoxide (DMSO) was purchased from Sigma (St. Louis, USA). Tamoxifen and celastrol were dissolved in DMSO (0.1%, *v*/*v*, final concentration), then sterilized by a 0.22 *μ*m pore filter (Merck Millipore, Bedford, USA). Antibodies were purchased from the following companies: LC3I/II, P62, phosphor-Akt, and phosphor-mTOR from Cell Signaling Technology (Danvers, UK); *β*-actin, anti-mouse-IgG, and anti-rabbit-IgG from Santa Cruz Biotechnology (Santa Cruz, CA, USA); and Hoechst 33342 from Guangzhou Ribobio (Guangzhou, China). The Annexin V-FITC Apoptosis Detection Kit and 3-(4,5-dimethylthiazol-2-yl)-2,5-diphenyltetrazolium bromide (MTT) were purchased from KeyGEN Biology Co. (Nanjing, China). Alexa Fluor 488 goat anti-rabbit-IgG (A11008) was purchased from Invitrogen (Carlsbad, CA).

### 2.3. Cell Viability Assay

Cell viability was measured by the MTT assay. Cells in the logarithmic growth phase were seeded in 96-well plates at a density of 1 × 10^3^/well and incubated for 24 h. Then, the cells were treated with different concentrations of celastrol (0.1, 0.3, 1, 3, and 10 *μ*M) and TAM (0.3, 1, 3, 10, and 30 *μ*M) and the combination of celastrol and TAM for 24, 48, and 72 hours, respectively. 50 *μ*l of the MTT reagent was added to each well and incubated for 4 hours at 37°C before the end of the incubation. Then, the culture medium was removed, and the purple formazan crystal was dissolved with 150 *μ*l DMSO. The resulting absorbance at 490 nm was determined by a microplate reader (iMark, Bio-Rad, Hercules, CA, USA). The 50% inhibitory concentration (IC50) was calculated according to the dose-response curve.

### 2.4. Analysis of Combined Drug Effects

The synergistic effect of celastrol in combination with TAM on MCF-7 cells was evaluated by isobolographic analysis. The IC50 values of tamoxifen alone and celastrol alone were plotted in the *X* and *Y* axes, respectively. The straight line connecting the two intercept points indicated an additive effect of two drugs. Then, we plot the values of the IC50-mix (TAM combined with celastrol). The points below or above the line indicated a synergistic or antagonistic effect, respectively.

### 2.5. Clonogenic Cell Survival Assay

MCF-7 cells were seeded at 100 cells/well in 6-well plates and dispersed evenly by shaking the dishes slightly. After attachment, cells were treated with either celastrol, TAM, or a combination of TAM+celastrol for about 7 days when the cells grew to visible colonies. The medium was discarded, and the cells were washed with PBS twice. The colonies obtained were fixed with 4% paraformaldehyde for 20 min and again washed twice with PBS followed by staining with 0.1% crystal violet. Finally, the dye was washed with PBS. The colonies with more than 50 cells were counted under an ordinary optical microscope.

### 2.6. Flow Cytometry Analysis

MCF-7 cells in the logarithmic growth phase were seeded in six-well plates and treated with celastrol (0.3 *μ*M), TAM (1, 3, and 10 *μ*M, respectively), and the combination for 24 hours. Then, the cells were harvested, rinsed with cold phosphate-buffered saline (PBS), and fixed with 75% cold ethanol solution at 4°C overnight. After fixation, the cells were washed twice and resuspended in 50 *μ*g/ml of propidium iodide (PI) solution containing 0.5% Triton X-100 and 2% RNase A. Cell cycle analysis was performed by flow cytometry (FACScan, Becton Dickinson, Mountain View, USA).

Apoptosis analysis was carried out by detecting phosphatidylserine (PS) externalization using flow cytometry. After treatment, the cells were resuspended at a concentration of 1 × 10^6^ cells/ml. Annexin V and PI were added at concentrations specified, according to the manufacturer's protocol. After 15 minutes of incubation at room temperature in the dark, the samples were available for flow cytometry analysis of apoptosis.

### 2.7. Western Blot Analysis

Cells were seeded in 60 mm dishes and treated with celastrol (0.3 *μ*M), TAM (1, 3, and 10 *μ*M, respectively), and the combination for 24 h. Then, the cells were harvested and lysed by RIPA buffer (Thermo Scientific). Protein lysates were size-fractionated on a 10% SDS-polyacrylamide gel and transferred onto PVDF membranes in Tris-glycine buffer (pH 8.4) containing 20% methanol. Membranes were blocked in 5% dry nonfat milk in Tris-buffered saline containing 0.1% Tween-20 (TBST) for 1 h at room temperature. After extensive wash, membranes were incubated with different specific antibodies overnight at 4°C. Then, membranes were incubated with appropriate secondary antibodies for 1 h at room temperature after 3 times of washing with TBST. Protein bands were visualized with enhanced chemiluminescence detection reagents (Thermo Scientific) and the Bio-Rad Gel Doc/ChemiDoc Imaging System (Bio-Rad, Hercules, USA). Data were analysed by Quantity One software (Bio-Rad).

### 2.8. Immunofluorescence Analysis

For immunofluorescence analysis, MCF-7 cells were seeded on coverslips and treated with different drugs for 6 h. Then, cells were fixed with 4% paraformaldehyde for 30 min at room temperature and subsequently permeabilized with 0.5% Triton X-100. After being washed with PBS, the cells were blocked in 5% BSA for 60 min, followed by overnight incubation with anti-LC3 antibody (1 : 100) described previously at 4°C. Afterward, the cells were washed with PBS again and incubated with Alexa Fluor 488 goat anti-rabbit IgG (1 : 500) for 2 h. The nuclei were counterstained with DAPI, and cells were observed under a fluorescence microscope (Becton Dickinson).

### 2.9. *In Vivo* Animal Model

Female BALB/c athymic nude mice were obtained from Nanjing Medical University Animal Center (Nanjing, China) at approximately 6 weeks of age and were maintained in a light (12 h/12 h light-dark cycle) and temperature-controlled (22-24°C) SPF environment with food (pelleted rodent chow) and water available ad libitum. This study was approved by the Animal Care Committee of Nanjing Medical University. Three mice were inoculated subcutaneously on the back with 5 × 10^6^ MCF-7 cells in 100 *μ*l. When the volume of the allograft tumours reached 500 mm^3^, the tumours were completely replicated and divided into tumour tissue pieces of 1 mm × 1 mm × 1 mm in the culture medium and transplanted into the right breast fat pad of 32 mice. After 20 days, when the average tumour volume reaches 110 mm^3^, the mice were randomly allocated to 4 groups. These were the vehicle (control, normal saline, 0.2 ml, *n* = 8, oral administration) group, the celastrol (0.5 mg/kg of celastrol, *n* = 8, oral administration) group, the tamoxifen (5 mg/kg of tamoxifen, *n* = 8, oral administration) group, and the combination (0.5 mg/kg of celastrol plus 5 mg/kg of tamoxifen, *n* = 8, oral administration) group.

After mice were sacrificed, tumour volumes and tumour weights were measured. Tumour weight/volume inhibition rate (TWIR/TVIR) = (1 − *T*/*C*) × 100%, where *T* is the average tumour weight or volume in the treatment group and *C* is the average tumour weight or volume in the control group.

Efficacy evaluation criteria: TWIR or TVIR ≥ 40%, and statistical analysis *P* < 0.05.

### 2.10. Statistical Analysis

Data were presented as means ± standard deviation (SD), representative of at least three independent experiments. All statistical analyses were performed using a one-way analysis of variance (ANOVA), followed by a *Dunnett post hoc* test, employing GraphPad Prism 6.0 software (GraphPad, San Diego, CA, USA) and R version 3.4.0 (http://www.r-project.org/). *P* < 0.05 was considered a statistically significant difference.

## 3. Results

### 3.1. Combination of Celastrol and Tamoxifen Resulted in Synergistic Cytotoxic Effects in MCF-7 Cells

**Figure 1:**
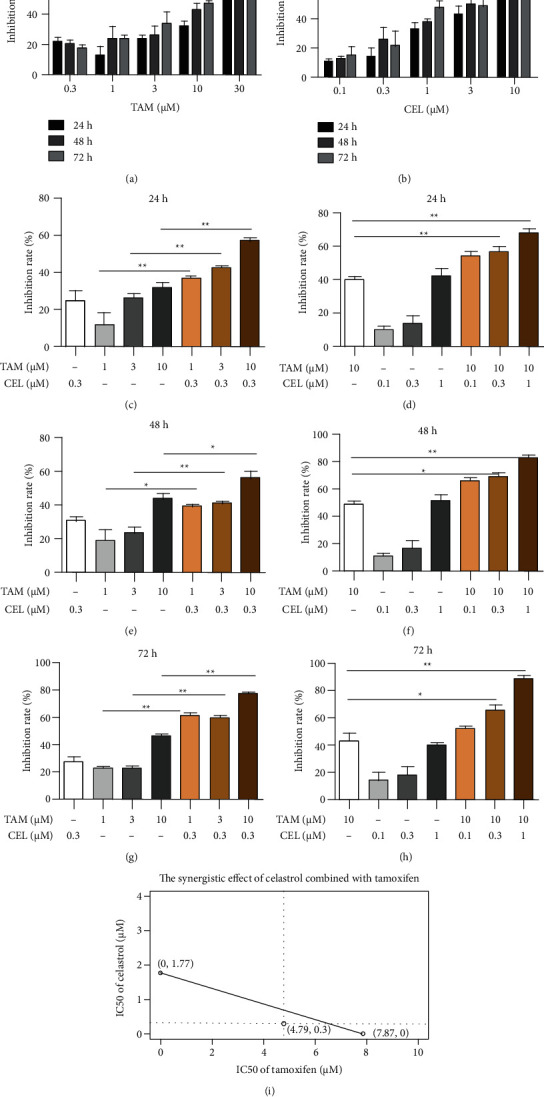
Effects on MCF-7 cell inhibition at different times and with different doses of TAM, celastrol, and their combination. (a–h) The effects on MCF-7 cell inhibition with different doses of TAM, celastrol, and their combination at 24 h, 48 h, and 72 h, respectively. (i) The IC50 values of celastrol and tamoxifen on MCF-7 cells were 1.77 and 7.87 *μ*M at 48 h, respectively. The IC50 value of tamoxifen was 4.79 *μ*M when combined with celxastrol (0.3 *μ*M). The empty dot below the line indicates a synergistic effect by isobolographic analysis.

To investigate the effect of celastrol combined with tamoxifen on the growth of MCF-7 cells, the cells were treated with various concentrations of celastrol (0.1, 0.3, 1, 3, and 10 *μ*M) or TAM (0.3, 1, 3, 10, and 30 *μ*M) for 24, 48, and 72 hours. Both celastrol and tamoxifen showed dose-dependent cytotoxic effects on MCF-7 cells, as shown in Figures 1(a) and 1(b). The IC50 values of celastrol and tamoxifen on MCF-7 cells were 1.77 and 7.87 *μ*M at 48 h, respectively. Then, celastrol at a concentration of 0.3 *μ*M was combined with different concentrations of tamoxifen, or tamoxifen (10 *μ*M) was combined with different concentrations of celastrol for 24, 48, and 72 hours. As shown in Figures 1(c)–1(h), the combination of the two compounds contributed to the enhanced cytotoxicity in MCF-7 cells compared with celastrol or tamoxifen alone. The IC50 value of the combination treatment was 4.79 *μ*M in response to 0.3 *μ*M celastrol, which was much less than that of tamoxifen alone. Moreover, an isobologram was plotted according to the IC50 values of celastrol, tamoxifen, and the combination. The results indicated that the combination of celastrol and tamoxifen exerted a synergistic cytotoxic effect in MCF-7 cells (Figure 1(i)).

### 3.2. Combination of Celastrol and Tamoxifen Inhibited the Colony-Forming Ability of MCF-7 Cells

**Figure 2:**
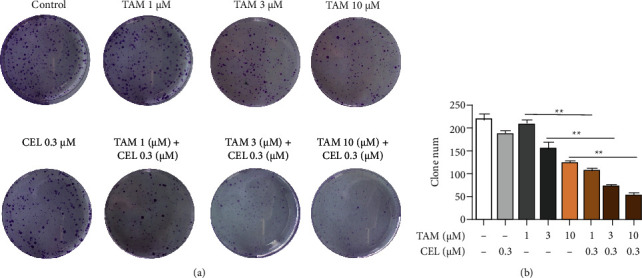
Combination of celastrol and tamoxifen inhibited the colony-forming ability of MCF-7 cells. Tamoxifen affects the colony-forming ability of MCF-7 cells in a dose-dependent manner, and this effect is more pronounced when combined with celastrol.

The effect of celastrol combined with tamoxifen on MCF-7 cell proliferation was evaluated by a colony formation assay. Both celastrol (0.3 *μ*M) and tamoxifen (1 *μ*M) treatment alone only slightly suppressed the number of colonies. Tamoxifen inhibited the colony formation of MCF-7 cells in a dose-dependent manner, and this effect was further enhanced by the combination treatment (Figure 2(a)). Statistical analyses of the colony formation studies revealed a synergistic inhibitory effect of the combination on MCF-7 cells (Figure 2(b)).

### 3.3. Celastrol Enhanced the Effect of Tamoxifen on Apoptosis in MCF-7 Cells

**Figure 3:**
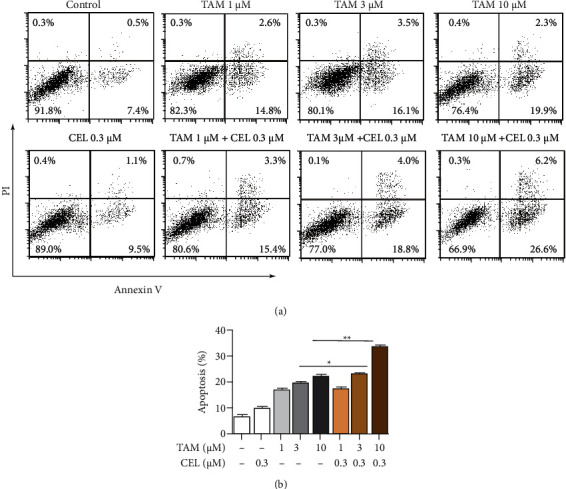
Effects of tamoxifen, celastrol, and the combination of tamoxifen and celastrol on apoptosis in MCF-7 cells. After treatment with celastrol (0.3 *μ*M), TAM (1, 3, and 10 *μ*M), and the combination for 24 hours, cell apoptosis was detected. The lower concentration of celastrol (0.3 *μ*M) did not increase apoptosis in MCF-7 cells; the medium-high concentration of TAM (3, 10 *μ*M) significantly increased the apoptosis rate of MCF-7 cells, and even the addition of celastrol at a lower concentration significantly increased cell apoptosis.

An apoptosis assay was performed to determine the mechanism of cell death induced by the combination treatment in MCF-7 cells. Cells were treated with celastrol (0.3 *μ*M), TAM (1, 3, or 10 *μ*M), or the combination for 24 hours. To further confirm the relationship between the combination treatment and apoptosis, apoptotic cells were detected by Annexin V-FITC/PI staining and flow cytometry analysis. The combination treatment significantly increased the apoptosis rate compared with tamoxifen alone in MCF-7 cells (Figure 3). The apoptosis rate of MCF-7 cells synergistically was enhanced up to 33.6% ± 1.217% by treatment with the combination of celastrol (0.3 *μ*M) and tamoxifen (10 *μ*M). This result suggested that celastrol enhanced the effect of tamoxifen on apoptosis in MCF-7 cells.

### 3.4. Combination of Celastrol and Tamoxifen Caused G1 Phase Cell Cycle Arrest in MCF-7 Cells

**Figure 4:**
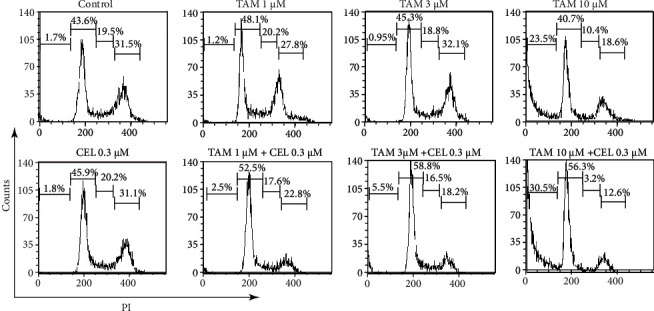
Effects of tamoxifen, celastrol, and tamoxifen and celastrol combinations on the cell cycle in MCF-7 cells. After treatment with the combination of tamoxifen and celastrol, the proportion of cells in the G1 phase was significantly increased compared with that of the control group, while the proportion of S phase and G2/M phase cells was reduced.

**Figure 5:**
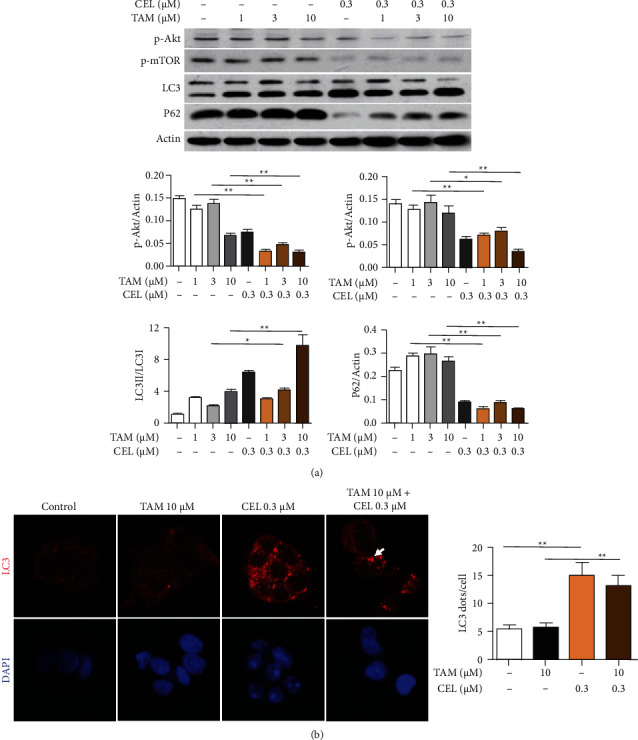
Combination of celastrol and TAM triggers autophagy in MCF-7 cells. (a) Protein levels of LC3, P62, p-Akt, and p-mTOR were detected by western blot after treatment with celastrol (0.3 *μ*M), TAM (1, 3, and 10 *μ*M), or the combination for 24 hours in MCF-7 cells. The protein expression of LC3II increased significantly, the ratio of LC3II/U increased (*P* < 0.05), and the protein level of P62, an autophagy substrate marker, was also significantly reduced in the combination group. In addition, the protein expression of p-Akt and p-mTOR was repressed in the combination group. (b) MCF-7 cells were treated with celastrol (0.3 *μ*M), tamoxifen (10 *μ*M) alone, or the combination for 6 h. Cells were fixed and double-labelled with an anti-LC3 antibody (red), and nuclei were stained with DAPI (blue). An increase in red punctate aggregation in the cytoplasm indicated the activation of autophagy in the celastrol group and combination group. The white arrow points to an autophagosome.

Since cell cycle progression is required for cell proliferation, the effect of the combination of celastrol and tamoxifen on cell cycle distribution in MCF-7 cells was investigated. The cells were treated with celastrol (0.3 *μ*M), TAM (1, 3, and 10 *μ*M), and the combination for 24 hours and stained with PI, and the cells were analysed by flow cytometry. As shown in Figure 4, the cells in the high TAM concentration group (10 *μ*M) showed a significant increase in the percentage of cells in the sub-G1 population (1.7% vs. 23.5%) compared with the cells in the control group. In the moderate-dose (1 *μ*M) and high-dose (10 *μ*M) groups, the percent of cells in the G1 population was increased when celastrol (0.3 *μ*M) was combined with TAM in the low-dose (1 *μ*M) group, while the cells in the S and G2/M populations were decreased compared with the TAM alone group (Figure 5). The results suggested that the combination of celastrol and tamoxifen caused G1 phase cell cycle arrest in MCF-7 cells.

### 3.5. Celastrol plus Tamoxifen Enhanced Autophagy in MCF-7 Cells

To determine whether the combination of celastrol and tamoxifen led to the activation of autophagy in MCF-7 cells, the protein levels of LC3I/II and P62 were analysed by western blot. MCF-7 breast cancer cells were treated with celastrol (0.3 *μ*M) or TAM (1, 3, and 10 *μ*M) in combination for up to 24 h. The protein expression of LC3II, an important component of the autophagosome, was significantly increased in the combination group, and the ratio of LC3II/LC3I was increased in the moderate and high concentration groups compared with the TAM treatment alone group (Figure 5(a), *P* < 0.05). Moreover, the level of P62, a substrate of autophagy, was dramatically decreased in combination treatment compared with tamoxifen alone (Figure 5(a)).

LC3 immunoreactivity analysis by immunofluorescence was then used to further examine the autophagy induced by the combination treatment. The cells were treated with celastrol (0.3 *μ*M), tamoxifen (10 *μ*M), or their combination for 6 h, and the nuclei were counterstained with DAPI. As shown in Figure 5(b), combination treatment altered the distribution of LC3, and coarser dots and punctate staining were formed in combination treatment than in tamoxifen alone. Statistical analysis was performed as follows: autophagic cells were defined as cells with five or more punctate LC3 dots, and the percentage of autophagic cells was assessed in 5 random fields. Distinct differences were found between the combination group and tamoxifen alone (Figure 5(b), *P* < 0.05).

### 3.6. Inhibitory Effect of Celastrol Combined with Tamoxifen on the Akt/mTOR Pathway in MCF-7 Cells

Based on the above results, the combination treatment increased apoptosis and induced autophagy in MCF-7 cells. Since the PI3K/Akt pathway plays an important role in the regulation of cell proliferation and death and Akt is located downstream of PI3K and is a main effector in the signaling pathway, the expression of p-Akt was detected to investigate whether the PI3K/Akt pathway is affected by combination treatment. MCF-7 cells were treated with celastrol (0.3 *μ*M), different concentrations of TAM (1, 3, and 10 *μ*M), and the combination for 24 h. Then, the activation level of Akt (p-Akt) was measured by western blotting assay. The expression of phosphorylated Akt was significantly lower in MCF-7 cells exposed to the combination treatment than in cells exposed to tamoxifen alone (*P* < 0.05), as shown in Figure 5(a), indicating a decrease in Akt activation.

To further confirm the above inhibitory effect, the level of p-mTOR was also detected by western blotting. The activity of mTOR, a downstream target of the PI3K/Akt pathway, is modulated by the pathway. The level of mTOR phosphorylation was significantly lower in the combination groups than in the tamoxifen alone group (*P* < 0.05), as shown in Figure 5(a), indicating a decrease in p-mTOR. These results indicated that the deactivation of Akt and mTOR may be beneficial for excessive autophagic cell death in MCF-7 cells.

### 3.7. *In Vivo* Effects of Celastrol Combined with Tamoxifen on MCF-7-Implanted Athymic Nude Mice

**Figure 6:**
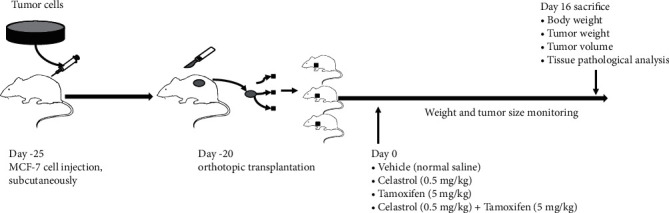
*In vivo* study design.

**Figure 7:**
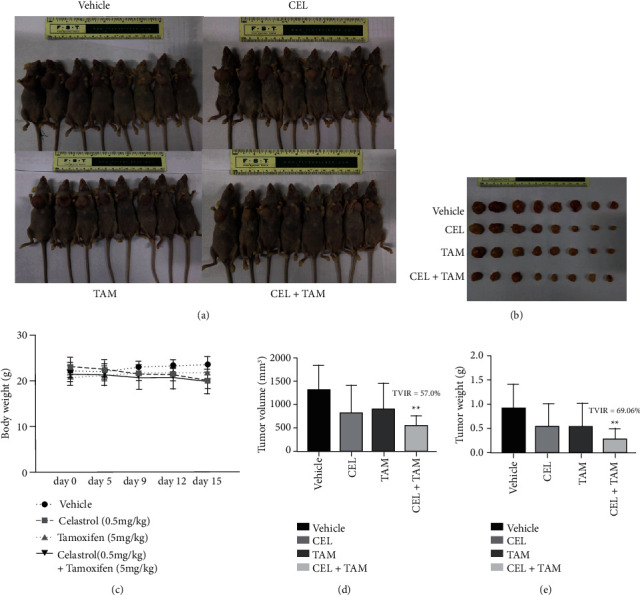
Effects of tamoxifen, celastrol, and the combination of tamoxifen and celastrol in MCF-7 tumour-implanted athymic BALB/c nude mice. The tumour-bearing mice were sacrificed on day 15. Representative photographs (a, b) of gross tumours in the mice in the vehicle, CEL, TAM, and CEL+TAM groups. Body weight (c), tumour volume (d), and tumour weight (e) were significantly decreased in the CEL+TAM group compared with the tamoxifen alone group.

To further assess the antitumour effects of celastrol combined with tamoxifen, female athymic BALB/c nude mice were used as *in vivo* models (Figure 6). All the mice were sacrificed on day 15, and the tumour volumes and weights were measured. The results revealed that the tumours in the combination group were significantly smaller than those in the tamoxifen alone group, with tumour volumes of 561.0 ± 206.75 mm^3^ and 880.37 ± 565.98 mm^3^, respectively, while the tumour weights were 0.29 ± 0.21 g and 0.58 ± 0.45 g, respectively (*P* < 0.05) (Figure 7). The tumour weight inhibition rate (TWIR) was 69.06%, which was greater than 40%, indicating that the combination of the two agents was effective.

**Figure 8:**
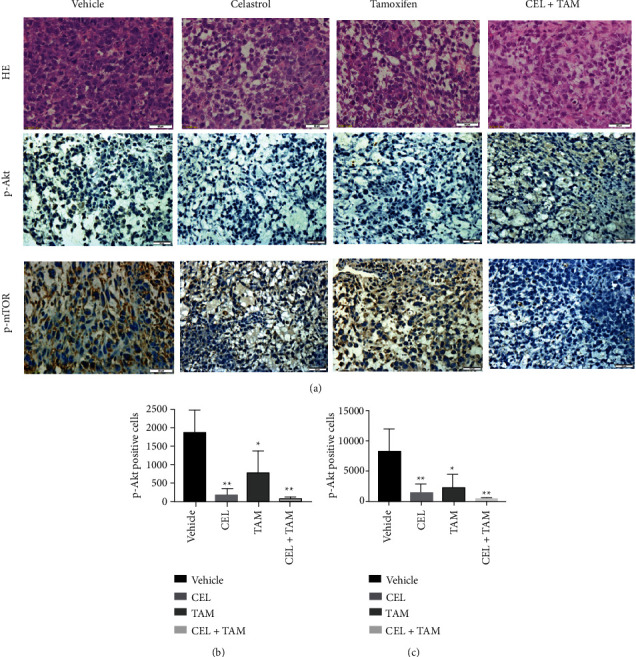
HE staining and p-Akt and p-mTOR detection by immunohistochemical analysis of tumour tissues. The phosphorylation of Akt and mTOR was significantly repressed in the combination group.

Immunohistochemical analysis indicated that, compared with that in the tamoxifen group, the phosphorylation of Akt and mTOR was significantly repressed in the combination group. As shown in Figure 8, there were very few p-Akt- and p-mTOR-positive cells in the combination group.

## 4. Discussion

Tamoxifen has been a highly successful adjuvant endocrine drug for over 30 years due to its availability, well-recognized safety in the clinic, and function; it can act as an antagonist through competition with oestradiol to bind to ER*α* and modulate gene expression in ER*α*-positive breast cancer cells [4, 5]. However, combination therapies with other synthetic and natural agents have been proposed to overcome the adverse effects of tamoxifen and drug resistance.

In this study, we investigated the synergistic effects of celastrol combined with tamoxifen on MCF-7 cells and an MCF-7-implanted animal model and explored the possible mechanisms. Our results demonstrated that tamoxifen induced apoptosis and autophagy in MCF-7 cells in a dose-dependent manner. Furthermore, celastrol exerted a synergistic effect with tamoxifen on MCF-7 cells. The anticancer effect of the combination treatment may depend on the inhibition of the Akt/mTOR signaling pathway. This study further verified the synergistic antitumour effects of these two drugs in an animal model.

Apoptosis, as a type-I programmed cell death (PCD) mechanism, plays a key role in a series of pathological processes, especially in cancer. The inhibition of cell apoptosis is involved in the development of drug resistance [16, 17]. Therefore, promoting apoptosis is often used as a potential anticancer strategy. Our results confirmed that apoptosis could be induced synergistically by the combination of celastrol and tamoxifen in MCF-7 cells.

Autophagy, an intracellular lysosomal degradation process, has been recognized as a type-II cell death mechanism by many researchers [16, 17]. This form of cell death plays a critical role in cellular homeostasis, mammalian development, and immunity. Growing evidence supports the prosurvival or prodeath effects of autophagy in tumour growth [18, 19]. Autophagy leads to a cytoprotective response and endoplasmic reticulum stress conditions involving nutritional starvation of amino acids or fatty acids, hypoxia, oxidative stress, damaged mitochondria, and chemotherapies. In contrast, autophagy can lead to cell death itself in some cellular contexts, either in collaboration with apoptosis or as a backup mechanism when apoptosis is defective [16, 20, 21]. Although the relationship between autophagy and apoptosis is still unclear, more studies have suggested that autophagy is presumably a target pathway of anticancer therapeutic agents [18, 22]. In advanced cancers, both enhancing autophagy and inhibiting it have been proposed as therapeutic strategies [23]. The vast majority of studies focused on inhibiting autophagy to prevent the renewal of cellular proteins and other molecules that help cancer cells survive under stressful conditions such as hypoxia and nutrient deprivation and to enhance other cancer treatments including chemotherapy and radiation [24]. However, some scientists focused on using natural compounds to activate overautophagy and cause cell death [22, 25, 26].

This study is aimed at observing the effect of autophagy induced by the combination treatment of celastrol and tamoxifen in MCF-7 cells. Microtubule-associated protein 1 light chain 3 (LC3), an important component of autophagy, appears as LC3I and LC3II in the cytoplasm. The conversion of LCI to LC3II through proteolytic cleavage and lipidation is considered a hallmark of mammalian autophagy. Therefore, the level of LC3II or the ratio of LC3II/LC3I was used to reflect the degree of cell autophagy [19]. It was found in this study that the combination of celastrol and tamoxifen could significantly enhance cell autophagy via LC3II accumulation. Moreover, the protein level of P62, a substrate of autophagy, was detected. Our results confirmed that the protein level of P62 was reduced dramatically, further indicating an enhancement of autophagy.

Given that both apoptosis and autophagy were found to be simultaneously induced in MCF-7 cells by the combination treatment of celastrol and tamoxifen, we sought to identify their relationship. We were interested in studying the PI3K/Akt/mTOR signaling pathway. PI3K/Akt is a major signal transduction pathway that plays a key role in the regulation of cell proliferation, survival, apoptosis, and autophagy [27]. The PI3K/Akt signaling pathway is frequently hyperactivated in many cancers. PI3Ks are a superfamily containing three classes, among which class I and III PI3Ks are involved in autophagy. Akt is a principal mediator in the PI3K/Akt signaling pathway, and mTOR is the downstream target of the PI3K/Akt pathway that is mainly regulated by the PI3K/Akt pathway. Numerous studies have shown that the process of autophagy is negatively regulated by activation of class I PI3K through upregulation of mTOR [28, 29]. In this study, the results showed that the expression of phosphorylated Akt (p-Akt) in MCF-7 cells exposed to combination treatment notably decreased compared with MCF-7 cells exposed to tamoxifen alone. It also showed that the expression of phosphorylated mTOR (p-mTOR) was dramatically reduced by the combination treatment. Our results showed that the combination treatment of celastrol and tamoxifen led to decreased p-Akt and p-mTOR in MCF-7 cells, along with increased expression of autophagy marker proteins, such as LC3II and P62, suggesting that cell autophagy induced by combination treatment was attributed to the repression of the Akt/mTOR signaling pathway. The study on MCF-7-implanted BALB/c mice also provided proof that compared with the tamoxifen alone group, the phosphorylation of Akt and mTOR was significantly repressed in the combination group. However, the exact mechanisms of the relationship between autophagy and apoptosis induced by the combination treatment of celastrol and tamoxifen in MCF-7 cells were not addressed in this study.

In conclusion, celastrol increased the sensitivity of MCF-7 cells to tamoxifen by inducing both apoptosis and autophagy. Induction of cell autophagy might be attributed to the inhibition of the Akt/mTOR signaling pathway. The combination of celastrol and tamoxifen exerts synergistic antitumour effects *in vivo* and *in vitro*. These findings support the possibility of the use of celastrol as a potential adjuvant drug or sensitizer for endocrine therapy in breast cancer. Although the decrease in p-Akt and p-mTOR might contribute to the autophagy induced by the combination treatment, the potential mechanisms underlying autophagy and crosstalk between apoptosis and autophagy remain to be further elucidated. In addition, our findings need to be further confirmed in models of breast cancer in the future.

## Data Availability

The datasets generated and analysed during the current study are not publicly available as the data also forms part of an ongoing study but are available from the corresponding author on reasonable request.

## Supplementary Materials

Supplementary Materials We consulted some references and carried out the preliminary test in the animal model and chose the concentration of TAM and CEL. The reason for choosing this concentration is that TAM or CEL at this concentration does not affect the food intake and body weight of mice (see supplementary drug toxicity test).
