# Supplementary figures and images for: The Synergistic Effects of Celastrol in combination with Tamoxifen on Apoptosis and Autophagy in MCF-7 Cells
Source: J Immunol Res. 2021 Jul 22;2021:5532269. doi: 10.1155/2021/5532269 (PMC8324338; doi:10.1155/2021/5532269)

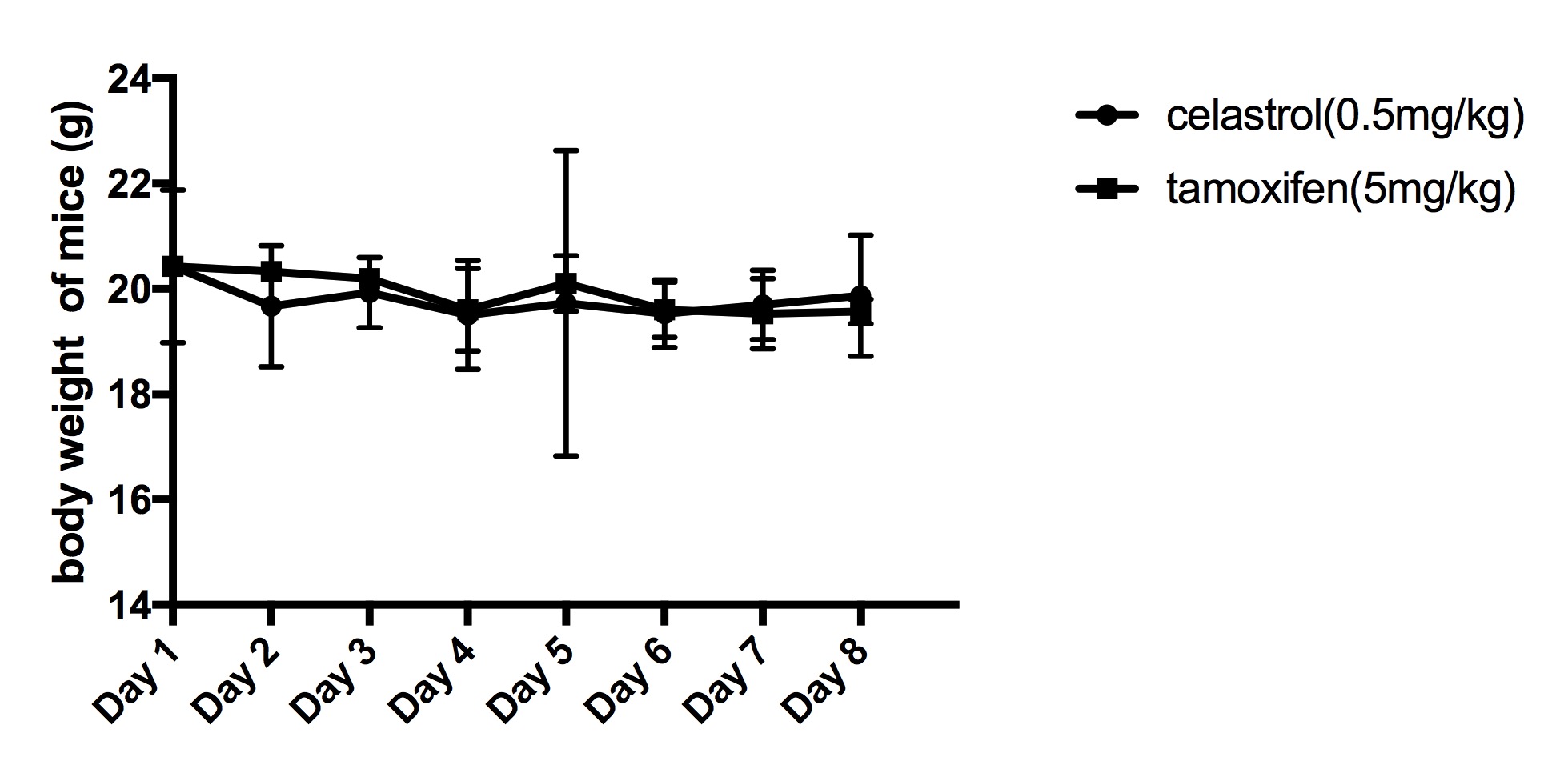


Supplement Drug toxicity test

Supplement: Supplementary Materials — We consulted some references and carried out the preliminary test in the animal model and chose the concentration of TAM and CEL. The reason for choosing this concentration is that TAM or CEL at this concentration does not affect the food intake and body weight of mice (see supplementary drug toxicity test). [file 5532269.f1.docx]
